# A measurement-based framework integrating machine learning and morphological dynamics for outdoor thermal regulation

**DOI:** 10.1007/s00484-025-02921-8

**Published:** 2025-04-21

**Authors:** Niloufar Alinasab, Negar Mohammadzadeh, Alireza Karimi, Rahmat Mohammadzadeh, Tamás Gál

**Affiliations:** 1 Department of Atmospheric and Geospatial Data Sciences, Faculty of Science and Informatics, University of Szeged, Szeged, Hungary; 2 Department of Architecture, Faculty of Art, Tarbiat Modares University, Tehran, Iran; 3 Department of Architecture and Urban Design, Faculty of Civil Engineering, University of Tabriz, Tabriz, Iran; 4 Institute of Building Materials, Building Physics, Building Technology and Design (IBBTE), University of Stuttgart, Keplerstr, Stuttgart, Germany

**Keywords:** Machine learning models, Outdoor thermal comfort, Morphological features, Bayesian optimization, Microclimate dynamics, SHAP values

## Abstract

This study presents a comprehensive investigation into the interplay between machine learning (ML) models, morphological features, and outdoor thermal comfort (OTC) across three key indices: Universal Thermal Climate Index (UTCI), Physiological Equivalent Temperature (PET), and Predicted Mean Vote (PMV). Based on a comprehensive field measurement for 173 urban canyons, proper dataset for summer outdoor thermal condition was provided. Concurrently, six distinct ML models were evaluated and optimized using Bayesian optimization (BO) technique, considering performance indicators like weighted accuracy, F1-Score, precision, and recall. Notable trends emerged, with the CatBoost Classifier demonstrating superior performance in UTCI prediction, the Random Forest classifier excelling in PET estimation, and the XGBoost Classifier achieving optimal PMV prediction. Furthermore, the study delved into the influence of morphological features on OTC, prioritizing factors using SHAP values. Results consistently identified 90-degree orientation, street width, and 180-degree orientation as pivotal factors influencing OTC, with varying degrees of sensitivity across different classifications of thermal stress. Analysis of binary SHAP values unveiled intricate relationships between urban features and OTC indices, emphasizing the critical influence of street orientation on regulating outdoor thermal environments for UTCI and PET scenarios. Surprisingly, street width emerged as the foremost influential factor within the PMV index, challenging established trends and highlighting the complexity of thermal comfort modeling. Additionally, current research delineates the multifaceted impact of street width on microclimate dynamics, enriching our understanding of urban thermal dynamics and emphasizing its role in mitigating thermal stress within urban environments.

**Supplementary Information:**

The online version contains supplementary material available at 10.1007/s00484-025-02921-8.

## Introduction

According to the United Nations, there is projected to be 6.4 billion people living in urban regions by 2050, which are more inhabited than rural regions (Feng et al. 2023). Urbanization provides more opportunities for social and economic advancement but triggers several environmental issues, among which urban overheating is particularly prominent (Falah et al. 2020; Huang et al. 2018). In environmental studies, considerable attention has been directed towards the Urban Heat Island (UHI) effect, a phenomenon characterized by elevated temperatures in urban areas relative to their surrounding suburban regions (Karimi et al. 2023). In addition to heightening energy demand in cities, this phenomenon also degrades air quality and increases the rate of heat-related illness and death (Adilkhanova et al. 2024; Farrokhi et al. 2024; Mohammed et al. 2024), thereby, urban areas experience harsh outdoor thermal environments (Dokhanian et al. 2023). Outdoor thermal comfort (OTC) is a complex concept shaped by numerous interrelated variables (Mohammad et al. 2021). In this regard, physiologically equivalent temperature (PET), the predicted mean vote (PMV), and universal thermal climate index (UTCI) are the models most frequently applied for assessing OTC (Lai et al. 2020). These indices encompass emotional, mental, and physiological factors that play a direct role on a person's outdoor thermal sensation (Zhang et al. 2024). On the other hand, indirect contributors of OTC include behaviors, personal traits, cultural elements, and geography (Halder et al. 2022; Zadeh et al. 2022). In this context, the following parts provide an in-depth description of methods for enhancing the outdoor thermal sensation of individuals.

Urban open areas exhibit diverse geometries, which significantly impact the urban thermal environment by altering radiation, wind speed, and heat transfer within these areas (Karimimoshaver et al. 2021). Therefore, street design emerges as a pivotal strategy in reducing the negative effects of climate change on urban areas, influenced by a range of urban geometry parameters, like the height of the buildings, width of streets, as well as the height to width ratio (H/W) and orientation of urban canyons (Nasrollahi et al. 2021). Overall, the effect of building height on OTC is contingent upon variables such as solar radiation and wind patterns. Similarly, the width of streets is a pivotal factor influencing outdoor thermal dynamics. Wider streets typically receive more solar irradiance, which can elevate daytime temperatures by increasing solar heat absorption (Ali-Toudert and Mayer 2007). Likewise, the proportional relationship between the width and height in urban canyons (H/W), plays a predominant role on providing optimal OTC. Compact urban areas with higher H/W ratio often experience decreased exposure to solar radiation (Lai et al. 2019), (Charalampopoulos et al. 2013), (Shih et al. 2017). Comparable findings were also reported in studies conducted in Malaysia (Qaid et al. 2016), Bangladesh (Kakon et al. 2009), and Taiwan (Lin et al. 2010). Furthermore, numerous investigations have approved the inverse correlation between H/W and mean radiant temperature (Tmrt) (Tan et al. 2013), (Krüger et al. 2011), (Wang et al. 2016). On the other hand, as wind interacts with buildings, its speed tends to decrease, particularly in areas with more enclosed urban forms (Taleghani et al. 2012), (Wang and Akbari 2014), (Yang et al. 2013), (Johansson 2006). Moreover, a parametric study employing Computational Fluid Dynamics (CFD) by (Yuan and Ng 2012) suggested that a decreased site coverage ratio generally enhances ventilation, contingent upon pedestrian-level building porosity.

The layout of streets, particularly their orientation, also plays a pivotal role in determining solar access of street and building surfaces, and even indoor area (Jamei et al. 2016), (Fouda 2014). Different street orientations result in varying degrees of shading and sun exposure throughout the day and across the seasons (Wang et al. 2024). Generally, NS streets offer more shade in summer but less in winter compared to EW streets (Fouda 2014), (Achour-Younsi and Kharrat 2016; Boukhelkhal and Bourbia 2016). In temperate, hot-humid, and hot-dry climates, EW orientation is typically deemed the least conducive to thermal comfort, with orientations closer to NS offering better conditions (Algeciras et al. 2016; Ali-Toudert and Mayer 2007; Bourbia and Awbi 2004). However, some studies present contrasting findings, suggesting EW as the optimal orientation based on Tmrt (Herrmann and Matzarakis 2012) and PET assessments (Abreu-Harbich et al. 2014). Furthermore, numerous studies have explored the influence of urban greenery on OTC. These studies have investigated aspects such as vegetation density and variations (Kim et al. 2022; Liu et al. 2022a, b), types of trees and planting proportions (Aminipouri et al. 2019; Karimi et al. 2020), canopy characteristics (Mahmoud and Abdallah 2022; Zadeh et al. 2022), and tree morphologies (Narimani et al. 2022). Regardless of the geography, additional vegetation has been shown to be a successful cooling technique worldwide (Chan et al. 2017; Chen and Ng 2013; Simon et al. 2018). By providing shading, trees improve OTC indices such as the PET (Karimi et al. 2020; Zadeh et al. 2022), PMV (Altunkasa and Uslu 2020; Rui et al. 2019), and UTCI (Li et al. 2020; Park et al. 2014). In this regard, the most often used metrics for thermal comfort in this respect are the PMV, PET, and UTCI (Lai et al. 2020). The PMV metric has been extensively utilized for investigating thermal comfort because of its condensed structure and thorough consideration of individual and environmental variables (Fanger 1970), (Hashemi et al. 2023). On the other hand, PET and UTCI are more commonly employed in the prediction of OTC since they extensively consider the outdoor environment (Fang et al. 2019; Potchter et al. 2018). Nonetheless, in order to addressing some individual differences and increase the accuracy of OTC analysis, researchers and technicians suggest employing data-driven approaches.

Recent studies have focused on using different machine learning algorithms, including the categorical boosting (Catboost), random forest (RF), decision tree (DT), k-nearest neighbour (KNN), support vector machine (SVM), and extreme gradient boosting (XGBoost), showing improvements in terms of prediction (Diz-Mellado et al. 2021; Guo et al. 2024a, b; Karimi et al. 2024; Niu et al. 2022; Wu et al. 2024; Xu et al. 2019). Different factors such as demographic categories, climatic conditions, and activity levels significantly influence OTC (An et al. 2021; Bassoud et al. 2021; Yao et al. 2022). Multiple ML methods have been evaluated by specialists to categorise the most suitable prediction models for specific scenarios and populations (Abdellatif et al. 2022; Lai and Chen 2019; Shan and Yang 2020). While ML applications for OTC have made significant strides, existing studies often exhibit limitations in terms of dataset comprehensiveness, interpretability, and relevance to real-world urban design. Many studies (Chai et al. 2020), (Fanger and Toftum 2002) rely on either single thermal comfort indices or limited ML models (Guo et al. 2024a, b; Jeong et al. 2022; Wang et al. 2023), restricting their ability to offer comprehensive design insights. Moreover, although several ML models have been applied to predict OTC indices, some studies (Morgan et al. 2023), (Xiao et al. 2024) often overlook the importance of interpreting the connections between OTC metrics and urban morphology, which is crucial for practical urban planning.

This study addresses these gaps, firstly by developing a high-resolution urban morphology dataset. A key influence of the current study is the construction of a comprehensive dataset covering 173 urban streets, including on-site measurements of crucial morphological features such as street orientation, width, and H/W ratios. This approach allows us to explore how morphological elements influence thermal comfort across varying stress conditions and climates. Moreover, a significant innovation of this study is the simultaneous analysis of three prominent OTC indices (UTCI, PET, and PMV) within one measurement framework. By simultaneously evaluating these indices, the study uncovers how different urban features affect each metric, enabling a deeper understanding of urban thermal dynamics. This multi-index approach provides a more robust prediction framework that caters to a variety of design scenarios and thermal stress levels. Furthermore, current study employs ML as a tool for insight, not just prediction. While several ML models have been applied to OTC prediction, our focus extends beyond mere model comparison. The current research, evaluates the performance of six prominent ML algorithms, using Bayesian optimization (BO) to fine-tune their parameters. However, the novelty lies in applying SHapley Additive eXplanations (SHAP) to the optimized models, offering a transparent, interpretable analysis of how morphological features influence each OTC metric. This interpretability advances the field by moving beyond black-box models, helping urban planners prioritize design interventions. Additionally, we highlight the varying sensitivities of thermal comfort metrics to morphological factors, suggesting that optimizing for one index may not always align with another. These nuanced insights offer practical guidance for urban planners, helping them make informed design decisions tailored to specific OTC goals.

To advance the field of outdoor thermal comfort, this study adopts a two-pronged approach: first, by incorporating three key OTC indices (UTCI, PET, and PMV) into a unified analytical framework, and second, by leveraging advanced machine learning techniques to enhance prediction accuracy and interpretability. In summary, this research concentrates on providing a comprehensive dataset that captures the complexity of urban morphology; integrating multiple OTC indices within a unified framework; leveraging SHAP to enhance the interpretability of ML models; and delivering actionable insights for designing thermally comfortable urban environments.

## Material and methods

**Fig. 1:**
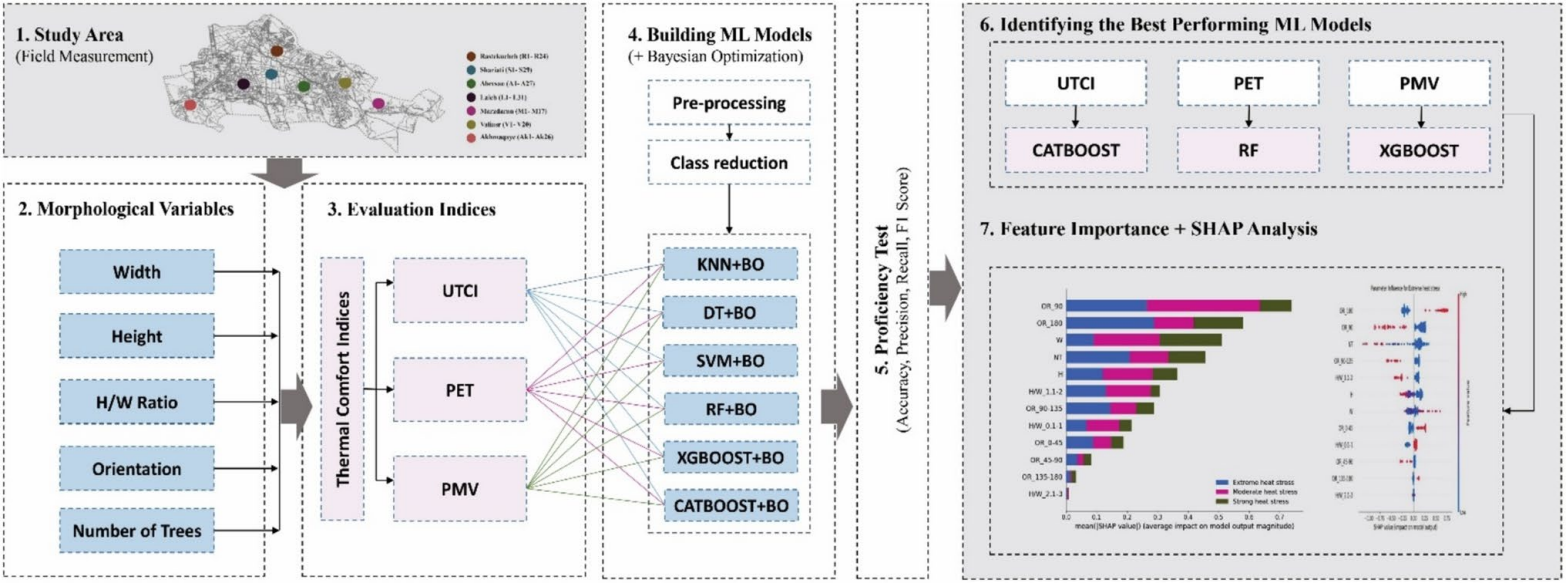
Research Diagram representing progress of study stages

The method employed in current research is structured into several key phases (Fig. 1), each aimed at systematically addressing the research objectives pertaining to OTC in urban environments. Initially, extensive on-site measurements were conducted across a diverse selection of one hundred streets, capturing various influential morphological factors affecting OTC. The collected data, including multiple thermal comfort metrics such as UTCI, PET, and PMV were categorized into three distinct research scenarios. This rich dataset formed the basis for subsequent analyses.

Following data collection, a rigorous data preprocessing phase was implemented to ensure the integrity and consistency of the dataset. This involved thorough cleaning of potential inconsistencies and outliers. Subsequently, a set of machine learning algorithms was implemented to represent the intricate connection between the morphological parameters and OTC measures. These algorithms encompassed both traditional techniques such as DT and KNN, as well as more advanced models like CatBoost, RF, XGBoost and SVM. To enhance the performance and predictive accuracy of the models across all three scenarios, BO was utilized to fine-tune the hyperparameters of the selected algorithms. Then, utilizing the preprocessed data, these algorithms were trained to develop predictive models for each of the three research scenarios. Finally, to facilitate the interpretation of the optimized models and discern the relative importance of various factors influencing OTC, the SHAP method was applied. This interpretative approach enabled the elucidation of the underlying mechanisms driving the predictive outcomes, thereby providing valuable insights into the factors contributing to outdoor thermal comfort in urban environments.

### Data collecting process

#### Case study

**Fig. 2:**
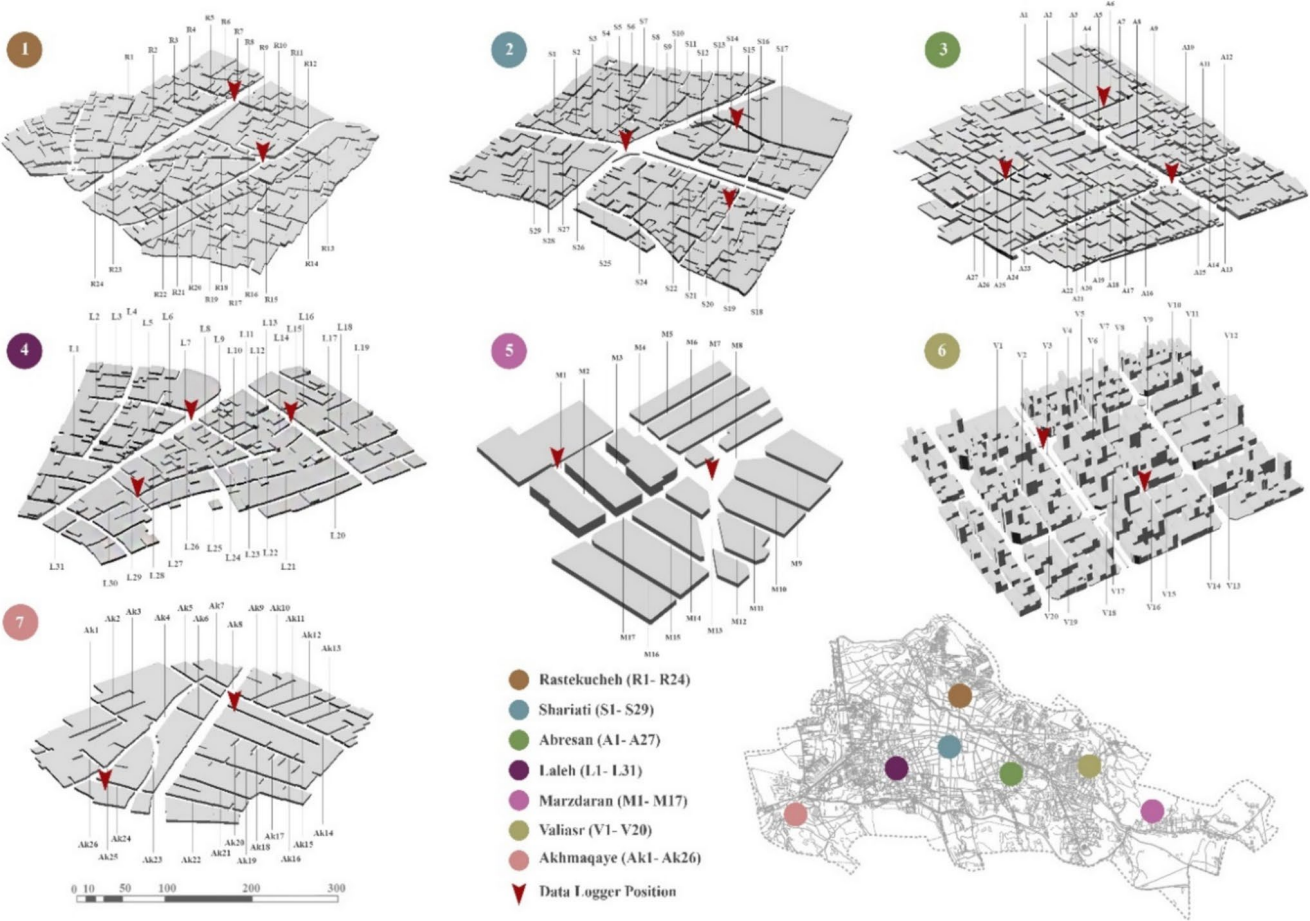
Study site locations and selected streets for measurement

Tabriz (46°17′39″ E, 38°4′9″ N), located in northwestern Iran, has a continental climate with distinct seasons influenced by its geography. As stated by the Köppen climatic category, the city falls within the cold semi-arid climate (BSk) class, marked by notable temperature variations throughout the year (Mohammadzadeh and Mohammadzadeh 2023). Summers in Tabriz are generally warm, with temperatures reaching their peak in July, the hottest month. The highest monthly average temperature (Ta) within this time frame is around 31.5 °C, with occasional spikes reaching up to 38.2 °C. Conversely, winters are cold and arid, with January being the coldest month. The lowest monthly average Ta in January hovers around − 2.3 °C, and temperatures can drop to as low as − 14.8 °C. An analysis of climate data spanning from 2012 to 2022 reveals the climatic nuances of Tabriz (Appendix 1). Annual average relative humidity in the city varies between 42.18% and 68.97%. This diversity in humidity levels contributes to the overall climate experience in Tabriz, providing a unique blend of weather conditions that shape the city's atmosphere (Zadeh et al. 2022). Over the past few years, the combined effects of urbanization and climate warming have further exacerbated heat stress, leading to a rise in frequency of heat waves in the area (Meresht et al. 2021). In fact, recent investigations suggest that UHI intensity in Tabriz exceeds 4 °C (Keikhosravi 2019). Between 1950 and 2017, Tabriz experienced a notable increase in both nighttime and daytime temperatures, with average rises of 0.24 °C and 0.45 °C over a decade, respectively (Rostamzadeh et al. 2021). To conduct a comprehensive study covering diverse sections of the city, a field experiment was conducted across various neighbourhoods. Initially, seven different types of neighbourhoods were identified as primary areas of focus. Subsequently, almost 25 streets within each neighbourhood were selected for detailed field measurements. The geographical locations of these neighbourhoods and the specific streets within them are illustrated in Fig. 2. The neighbourhoods under consideration represent diverse contexts within Tabriz, each offering unique morphological characteristics and socio-economic backgrounds.

First neighbourhood (Rastekucheh) is located in the historical context of Tabriz, with traditional landmarks and sites, which was registered in the UNESCO’s world heritage list. Buildings of this context are typically low-rise, and streets maintain their original narrow layout. The second neighbourhood (Shariati) occupies in a semi-historical context characterized by a mix of architectural styles and street designs. In contrast, the third (Abresan) and fourth (Laleh) neighbourhoods, situated in the eastern and western parts of Tabriz respectively, reflect an older but not necessarily historical ambiance. Here, buildings tend to be more recent, and streets are wider compared to their historical counterparts. Fifth neighbourhood (Marzdaran) embodies the contemporary face of the city with high-rised buildings and wide streets. Similarly, the sixth neighbourhood (Valiasr) aligns with modern urban planning principles, featuring structured building layouts and wide streets. Lastly, the seventh neighbourhood (Akhmaqayeh) represents an area with informal settlements and lower economic status. Buildings here are typically one to two floors high, and the streets maintain a narrow configuration. Figure 2 illustrates seven study area and selected streets for measurement purpose.

To ensure the acquisition of comprehensive data, our selection process for streets within each neighbourhood aimed to encompass a diverse range of characteristics. This involved considering various factors such as street orientation, width, and building heights. Given that the surrounding air temperature (Ta) can be affected by the local environment in a central range of 10–150 m, measurement points were strategically positioned at intervals of 15 m along each selected street. In total, 17 data loggers were deployed to record microclimate data and overlapping with morphological aspects, and finally 1168 points were identified. For each measured space, we meticulously recorded morphological characteristics, including the H/W, orientation, street width, building elevation, and the number of trees. This systematic approach ensured a thorough examination of each area's unique attributes. Additionally, to manage the abundance of data and streamline their interpretation, different orientations have been classified into six classes with 45-degree intervals (0–45°, 45–90, 90, 90–135, 135–180, and 180). Similarly, H/W ratios have been grouped into three classes based on predominant data (0.1 to 1, 1.1 to 2, and 2.1 to 3). Furthermore, the width of streets ranges from 2 to 38 m, and the elevation of buildings differs from 1 to 8 floors across different locations. Table 1 demonstrates predominant site configurations for each study area.

**Table 1:** Descriptions of site configurations

Area	Streets	Measurement Points	Number of Trees	H/W	Floor	Orientations	width(m)
1	R1- R 24	152	43	0.1–1, 1.1–2, 2.1–3	2–3	0–45, 45–90 90, 90–135, 180	2–28
2	S 1- S 29	210	339	0.1–1, 1.1–2, 2.1–3	2–4	0–45, 45–90 90, 135–180, 180	4–36
3	A 1- A 26	158	26	0.1–1, 1.1–2	1–3	0–45, 90–135	4–16
4	L 1- L 31	152	32	0.1–1, 1.1–2	1–3	0–45, 90, 90–135 135–180, 180	4–20
5	M 1- M 17	120	59	0.1–1, 1.1–2	2–8	0–45, 90–135 135–180, 180	6–38
6	V 1- V 20	210	68	0.1–1, 1.1–2	2–4	90, 180	6–32
7	Ak 1- Ak 26	166	13	0.1–1	1–2	45–90, 90, 135–180	2–12
Total	173	1168	-	-	-	-	-

#### Measurement procedure

The current research entailed micro-climate measurement and site monitoring during the summer months of July and August in 2022 in Tabriz, including two extreme heatwaves in mid- July and August 15 th to 21 st. The experiment extended over 62 days, with a systematic recording of meteorological parameters carried out in seven mentioned neighbourhoods from 11:00 A.M to 6:00 P.M. each experiment day. Meteorological measuring points were strategically positioned at interconnection spots of both wide and narrow streets to ensure comprehensive coverage. The placement of data loggers was determined to ensure optimal coverage across the study areas. All measurements were conducted simultaneously on the same days and times to account for potential climate variability. Additionally, in order to record accurate outcomes, measurements were systematically conducted at one minute intervals. It is significant to mention that in this study, the instruments complied with ISO 7726 criteria, certifying both prompt answer times and higher precision (Iso 2001). Detailed specifications of the measuring instruments used are outlined in Appendix 2. Among the four necessary meteorological parameters, direct measurements are feasible only for air temperature (Ta) and relative humidity (RH). However, calculating mean radiant temperature (Tmrt) and wind speed (V) is required for the remaining parameters. The most precise method for determining Tmrt involves measuring shortwave and longwave radiation fluxes in three dimensions (Thorsson et al. 2007). However, this approach necessitates expensive radiometers. A widely used alternative is to gauge Tmrt by measuring globe temperature (Johansson et al. 2014), which is more cost-effective due to the affordability of globe thermometers. In this effort, we opted for the latter method, obtaining Tmrt using the equation provided (Karimi and Mohammad 2022). Where D is the globe diameter (150 mm) and ε represents the globe emission (0.95).

1 $${T}_{MRT}= {\left[{\left({T}_{GLOBE}+273.15\right)}^{4}+\frac{1.1*{10}^{8}*W{s}^{0.6}}{{\varepsilon *D}^{0.4}}*({T}_{GLOBE}-{T}_{A})\right]}^{0.25}-273.15$$

To determine PET, wind speed (V) needs to be obtained at 1.1 m, whereas for UTCI, it should be recorded at 10 m. However, since V is rarely measured at these exact heights, it has been proposed to estimate the required V at x meter (*Vx*) using the log wind profile (Bröde et al. 2012):

2 $$Vx=Vm\times \frac{\text{log}(\frac{x}{z0 })}{\text{log}(\frac{m}{z0 })}$$

Which *Vm* refers to wind speed at the measured elevation, m shows measurement height (meter) and *z*_*0*_ = 0.01 m for an urban path (Havenith et al. 2012). In addition to environmental factors, the computation of PET and UTCI must also consider interior heat generation and clothing insulation. Interior heat generation rates were standardized at default amounts for PET (80 W) and UTCI (135 Wm- 2). The RayMan model adopts the 0.9 clo as a typical clothing insulation, while in BioKlima 2.6, clothing insulation isn't a necessary input as it's determined by a clothing model (Fröhlich et al. 2019; Havenith et al. 2012) integrated into the UTCI-Fiala method (Bröde et al. 2012). On the other hand, PMV has been adapted for outdoor conditions, known as"Klima-Michel-Modell (Kumar and Sharma 2020). This adaptation involves utilizing climatic data as input file, integrating short and longwave radiations, and considering default outdoor activities and wearing conditions (Jendritzky and Nübler 1981). Using the ASHRAE thermal perception scales, PMV forecasts the average reaction of a large number of people. In this regard, ASHRAE standard 55 and ISO 7730 are the guidelines that have incorporated the PMV model (Olesen and Brager 2004).

### Process of machine learning model establishment

Initially, the output dataset was categorised by employing pre-processing stage. After cleaning outliers and classification, several predominant ML algorithms was chosen as the main methods, including KNN, DT, SVM, RF, XGboost, and Catboost. To address potential class imbalance and improve model robustness, sample weights were calculated based on the distribution of classes in the training set. In the subsequent phase, we embarked on Bayesian optimization (BO), a method aimed at refining the hyperparameters of our initial machine learning models. This intricate process involved defining variable ranges for different algorithms including maximum depth, learning rate, and minimum sample size. By harnessing BO, we sought out the most effective variable arrangement within this hyperparameter area, with the overarching goal of maximizing cross-validation accuracy through a rigorous fivefold cross-validation approach. With our optimized parameters in place, we proceeded to construct six multi-class machine learning models. Python 3.9 was employed for this investigation, and all of the activities were carried out and documented primarily using the Jupyter Notebook platform. These models, finely tuned to reflect the best hyperparameters, were then subjected to analysis using the SHAP model. Through this analytical framework, we meticulously examined the influential characteristics shaping the outcomes of UTCI, PET, and PMV across varying morphologies. The insights gleaned from this analysis shed light on both the positive and negative impacts of individual features, offering valuable explanations for the model's predictions. Furthermore, this process provided a solid foundation for further optimization endeavours. Detailed insights into these methodologies are elaborated upon in the subsequent sections.

#### Data pre-processing and classification

Data preprocessing involves standard data cleaning techniques and normalization, which are crucial for identifying the most pertinent data for machine learning algorithms. In this regard, one-hot encoding for categorical variables is implemented and ensured that any missing values were either imputed or excluded based on their frequency and relevance in the dataset. Any outliers like the inaccuracies in data collecting process, null values, and out-of-range data, were removed during this stage. Subsequent to the primary cleaning process, the dataset consisted of 1168 data points. For this study, a random division approach was employed, where 70% of the dataset was allocated to the training set, and the 30% served as the testing set. To ensure potential class imbalance in the OTC index samples and prevent model bias, class weight adjustments is applied during model training. Specifically, the compute_sample_weight (class_weight ='balanced', y = self.y_train) function is impelented from sklearn.utils.class_weight, which dynamically assigns higher weights to underrepresented classes and lower weights to overrepresented ones.

The subsequent step prior to constructing the machine learning model involves categorizing the target columns, which signify the comfort level of each sample based on the thermal comfort index. To achieve this, each OTC index was classified into appropriate sub-classes. Decision of classification helps address the inherent uncertainty in predicting continuous values due to variations in environmental and morphological factors. Furthermore, classifying OTC indices improves the interpretability of model results, allowing for clearer communication of thermal comfort levels to urban planners, architects, and policymakers. From a model performance standpoint, classification tasks benefit from focusing on distinguishing between predefined comfort levels. This structured approach reduces the risk of overfitting to specific numeric ranges, leading to better generalization when applied to new, unseen data. Furthermore, classification allows us to optimize decision boundaries between comfort categories, which can improve the model’s predictive accuracy in real-world scenarios.

In this study, OTC indices such as the UTCI, PET, and PMV were utilized as the foundation data for training the machine learning models. These indices were calculated based on data collected from field measurements conducted during the experimental phase. The labeling of each sub-class strictly follows the original classification ranges defined in the respective scales (PET’s 9-level scale and UTCI’s 10-level scale, as referenced in (Fang et al. 2018), and for PMV, the ASHRAE Standard 55 and ISO 7730 guidelines (Olesen and Brager 2004). Due to the typically warmer environments experienced during summer days, the data range often exceeded the comfort level. Consequently, data falling below the comfort range was potentially removed from original scaling. Additionally, a few instances of out-of-range data for the UTCI index were identified and eliminated during the preprocessing stage. As a result of these adjustments, the original classifications of all three OTC indices were transformed into a 3-class category, as illustrated in Table 2.

**Table 2:** Dataset classification and feature weights for UTCI, PET, and PMV

Indices	Numerical Range	Class Name	Feature Weight
UTCI	+ 26 to + 32	Moderate heat stress	28%
	+ 32 to + 38	Strong heat stress	32%
	+ 38 to + 46	Very Strong heat stress	40%
PET	+ 29 to + 35	Moderate heat stress	21%
	+ 35 to + 41	Strong heat stress	20%
	> + 41	Extreme heat stress	59%
PMV	+ 2 to + 3	Warm	28%
	+ 3 to + 4	Hot	22%
	> + 4	Very Hot	50%

Regarding the dataset structure, Table 2 also presents a detailed breakdown of the sample distribution across different thermal stress levels for UTCI, PET, and PMV, including their feature weight percentages. Accordingly, given the potential disparity in sample distribution across categories, with some having significantly more samples than others, the Weighted-average evaluation technique adjusts for the imbalances. This ensures an equal assessment of efficiency and better addresses class inequity.

#### ML optimization process

The ML techniques chosen for this research encompass KNN, DT, SVM, RF, XGBoost, and CatBoost (G. Guo et al. 2003), (Kingsford and Salzberg 2008), (Noble 2006), (Breiman 2001), (Chen and Guestrin 2016), (Prokhorenkova et al. 2018). Appendix 3 comprehensively highlights the distinctive attributes of the algorithms and their definitions. In the next stage, Hyperparameter optimization involves finding the best configurations for individual machine learning models, using BO methodology with a Gaussian Process-based surrogate model. Unlike the traditional GridSearch approach, Bayesian optimization incorporates prior distributions of the objective function and iteratively explores the hyperparameter space to find the best solution. In fact, the BO process aims to find the best combination of hyperparameters that maximizes the performance of the model. The optimization process is configured with an acquisition function of Expected Improvement (EI), which effectively balances exploration and exploitation. The BO process systematically explored the search space for each ML model by iteratively selecting hyperparameter configurations that improved predictive accuracy. Initial hyperparameter distributions are defined based on prior domain knowledge and preliminary experimental results, ensuring a guided yet flexible search space. Key hyperparameters, such as the learning rate, tree depth, and number of estimators, are tuned using BO. A log-uniform distribution is employed for the learning rate to capture better variations at lower values and a uniform distribution for tree depth to prevent excessive complexity. The optimization process continues until a convergence criterion is met for each ML model. These selections enhance the robustness of the models while preventing overfitting.

In general, This approach promotes a more intellectually rigorous exploration of hyperparameters, reducing unnecessary experimentation and improving the efficiency of discovering the most suitable hyperparameter combinations (Snoek et al. 2012). Appendix 4 highlights various algorithms combined with BO and the resulting optimal hyperparameters for each OTC index.

#### Model performance

During the summer season, individuals are more likely to experience heat discomfort compared to cold sensations. Therefore, accurately identifying those who are susceptible to heat discomfort becomes crucial. In this context, OTC metrics play a vital part in identifying the appropriate thermal range in various locations. Utilizing OTC metrics as targets for machine learning models can lead to greater benefits, as it enables the models to effectively predict and classify individuals who may be at risk of experiencing heat discomfort. By leveraging OTC metrics in this manner, interventions and mitigation strategies can be targeted more precisely, thereby enhancing the overall effectiveness of heat stress management efforts.

In order to mitigate possible inequalities in categories, ML algorithms that show improved performance are selected for each OTC index. The evaluation of these models'performance metrics employs a weighted average method. This method involves calculating the weighted average of performance metrics like accuracy, precision, F1-score, and recall, for each category. The values allocated to each group are determined by the amount of samples within it. Also, in order to optimize classification performance, BO is employed for hyperparameter tuning, ensuring that the ML models were fine-tuned for each OTC index. For each OTC index, BO optimizes key hyperparameters to ensure that each model achieves the highest possible classification accuracy. Appendix 5 represents the definitions and equations used to determine all performance metrics within each category, including Accuracy, Precision, Recall, and F1 Score.

## Results

### Optimization and best model selection

To enhance prediction capabilities for each of the three scenarios, six distinguished ML algorithms were optimized by using BO method. Before doing formal hyperparameter space experiments, a number of exploratory examinations were carried out to ascertain the hyperparameter range for each ML algorithm. Table 3 presents the performance metrics of models across the three OTC units. The metrics include weighted accuracy, recall, precision, and F1-Score.

**Table 3:**
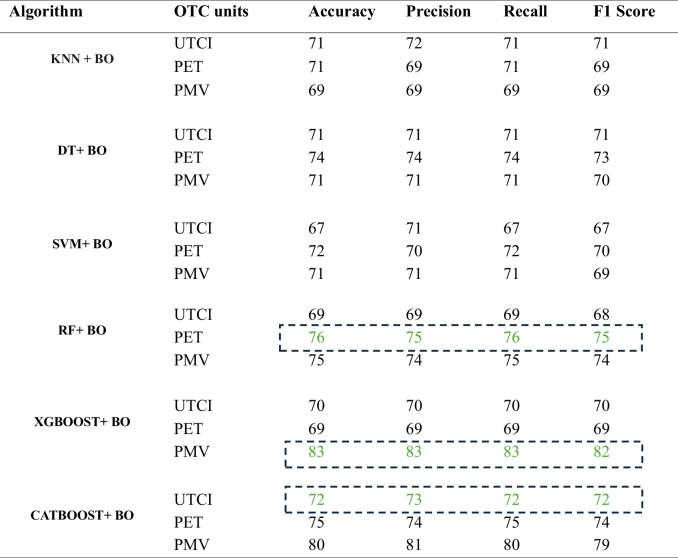
Performance metrics of six ML models + BO for UTCI, PET, and PMV

In general, marginal differences were observed in the performance of the six ML models concerning all three OTC indices. Notably, for the scenario focusing on UTCI, the CatBoost + BO stands out as the best-performing model across the entire dataset for this target, exhibiting the accuracy and F1 score of 72%. Conversely, the SVM model exhibited the lowest levels of accuracy and F1 score in this scenario, recording figures of 67% and 66%, respectively. Focusing on PET as the primary target, RF + BO emerged as the superior model, demonstrating an accuracy of 76% and an F1-Score of 75%. Conversely, KNN + BO displayed the lowest levels of accuracy and F1 score, registering at 71% and 69%, respectively. The XGBoost Classifier combined with BO (XGBoost + BO) emerged as the best model for the scenario, focusing on PMV as the primary target variable. This model displayed an accuracy of 83% and an F1-Score of 82%, consolidating its efficiency in this context. On the contrary, The K-Nearest Neighbours (KNN) algorithm + BO has demonstrated the lowest level of accuracy and F1 score, both registering at 69%, for the PMV unit. Table 2 comprehensively represents the performance metrics and the optimal model for each scenario.

### Model interpretation

#### Features importance rankings

For each of the three scenarios in the current research, feature importance ranking using SHAP value technique is employed. In Fig. 4, The average SHAP values for each morphological factor is represented on the horizontal axis, which shows the influence of the input variables on the model's output. Features with higher numerical values exert a more substantial influence on the model. For every OTC metric, twelve morphological features have been prioritized based on their importance and different colours correspond to different classification labels.

**Fig. 3:**
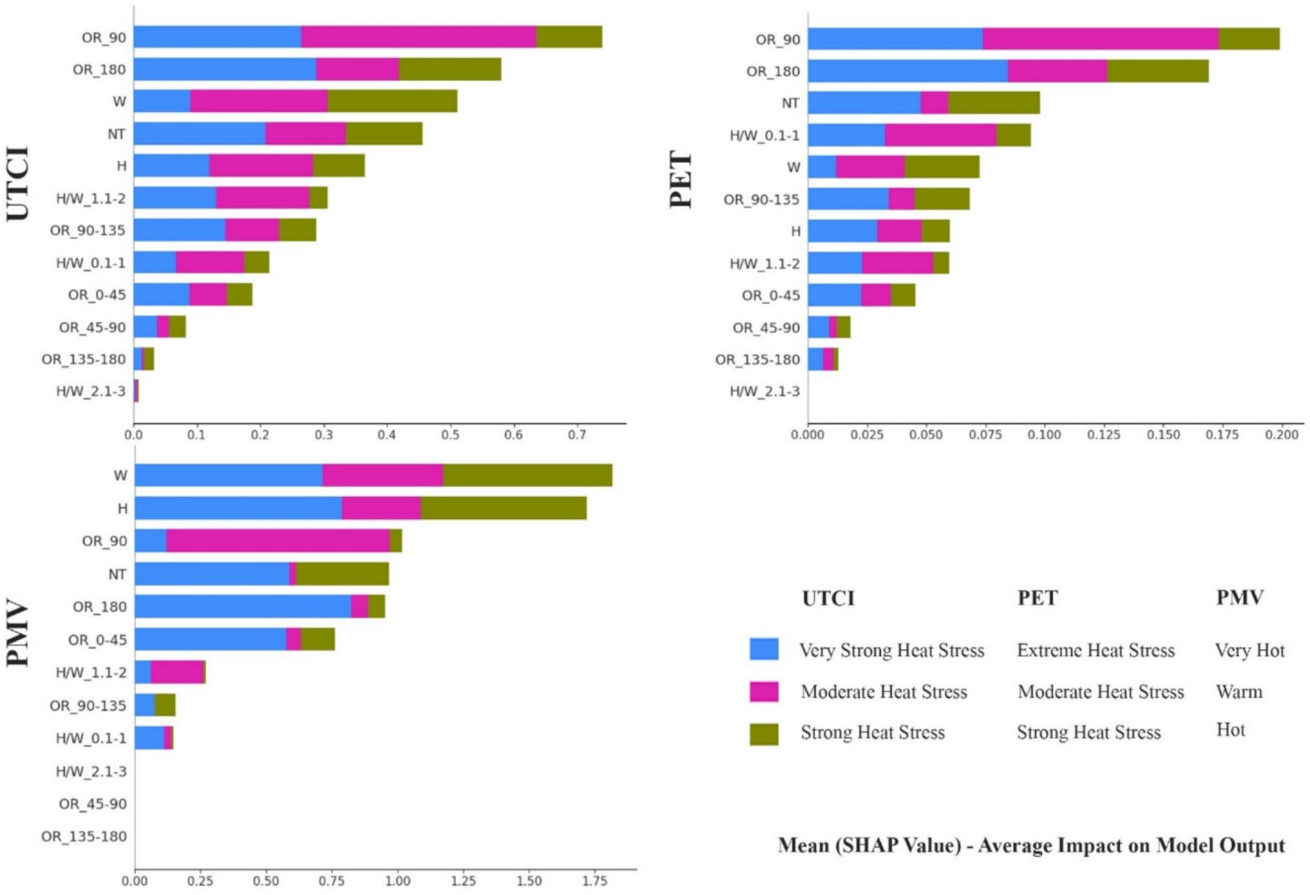
Classified Feature Importance of Different morphological factors for UTCI, PET, and PMV

According to the findings, for the UTCI index, the 90-degree orientation has the most important effect on the OTC. Following that, the 180-degree orientation, width, and number of trees are the subsequent major influencing factors, respectively. Based on Fig. 3, It is noteworthy that for the “Moderate heat stress"classification, the influence of the 90-degree orientation is the most pronounced, while for the"strong heat stress"classification, width emerges as the most influential factor, and for the"very strong heat stress"classification, the 180-degree orientation demonstrates the highest influence. In this regard, the 180-degree orientation, representing an East–West alignment, can have a significant impact on airflow and heat dispersion in urban areas. Streets aligned in this direction may disrupt prevailing wind patterns, potentially reducing the natural ventilation of the area. This can lead to stagnation of air, which hinders the dispersion of heat and pollutants, ultimately exacerbating thermal discomfort and contributing to poor air quality. During extreme heat stress, the inability to disperse heat efficiently results in the accumulation of heat in the street canyon, raising temperatures and increasing exposure to heat stress. Similarly, the lack of airflow can cause pollutants, such as particulate matter, to concentrate, further diminishing air quality. This disruption of both airflow and pollutant dispersion is a key factor that makes the 180-degree orientation a significant contributor to adverse thermal conditions.

When considering the PET index, akin to the UTCI, the 90-degree orientation stands out as the most influential aspect in OTC. Among the top five features, in addition to the significance of the 180-degree orientation, width, and number of trees, the role of the H/W ratio is noteworthy. Comparing different classes, for the"Moderate heat stress"class, the impact of the 90-degree orientation is most pronounced, describing that N-S oriented streets provide better thermal circumstances. Whereas, for the"Strong heat stress"and"Extreme heat stress"classifications, the 180-degree orientation emerges as the most important factor. This result also shows that streets aligned in this direction may interfere with the natural flow of wind, reducing ventilation in the area. This can cause the air to become stagnant, slowing the spread of heat and pollutants, which increases thermal discomfort and worsens air quality.

For the PMV index, there appears to be a deviation in feature rankings compared to previous indices. In this index, the Width of the street emerges as the most influential factor. However, despite differences in the ranking levels, the top five influential features remain consistent. Comparing different classes reveals that for the"warm"classification, the 90-degree orientation plays the most important role, while for the"hot"classification, width emerges as the most influential factor and for the"very hot"classification, 180-degree orientation demonstrates the greatest influence. Despite minor variations in numerical values and overall ranking, it is notable that classifications across all three indices under three different machine-learning models exhibit the same feature importance rankings.

#### Positive or negative effects of features

In order to analyse the positive and negative impact of each factor on different classifications, binary SHAP values are conducted which “Red” signifies larger feature values, whereas “Blue” is revealing of smaller feature values. Moreover, a negative SHAP value defines that the factor negatively influences thermal comfort, while a positive value specifies a positive impact. The legend located on the right side of the figures outlines the relationship between color gradients and the allied parameters.

**Fig. 4:**
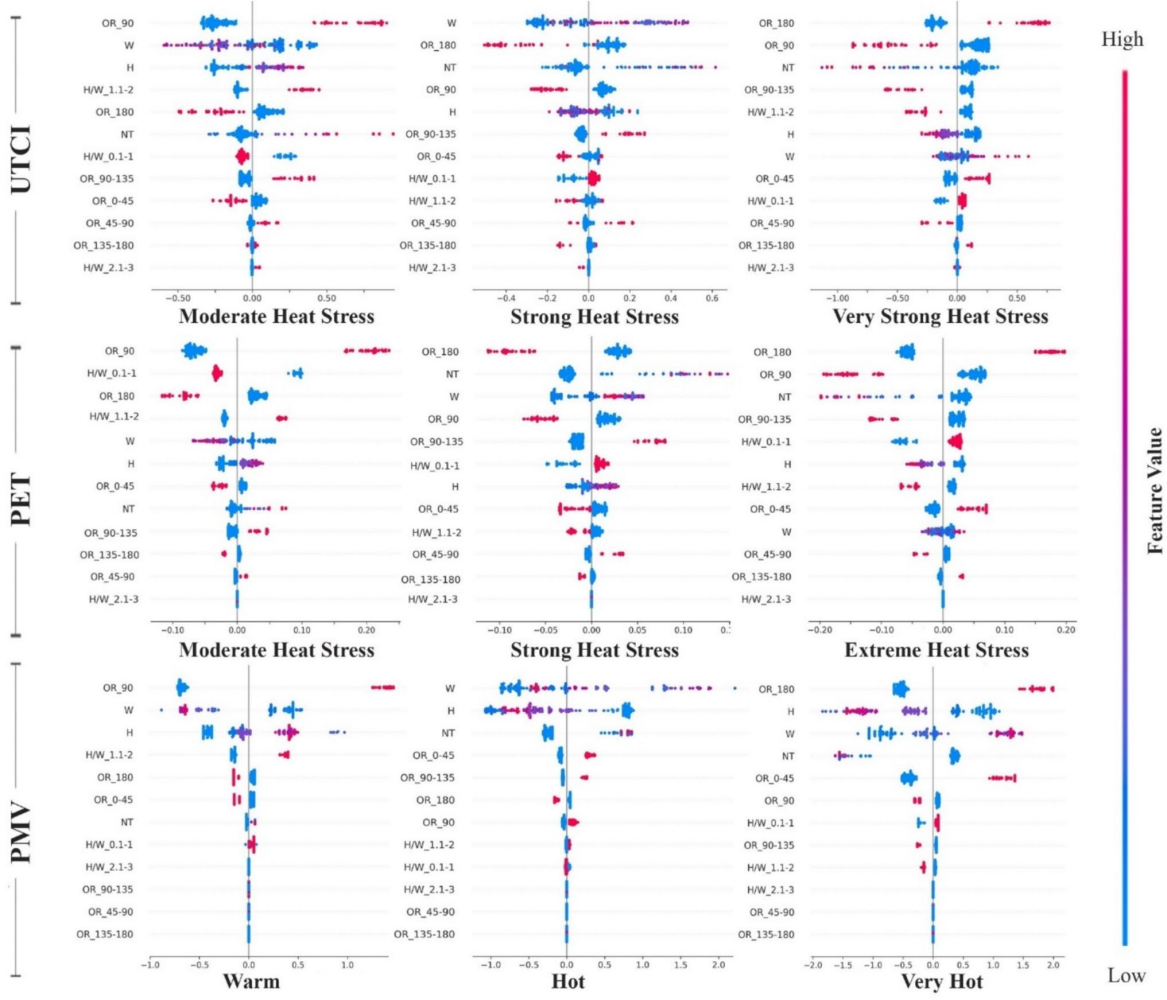
Positive and Negative impact of different features on UTC, PET, and PMV Classifications

For the UTCI index, Fig. 4 illustrates that in the"Moderate heat stress"classification, with the numerical range of + 26 to + 32; 90-degree orientations exert a notably positive impact as the most influential feature. This implies that streets oriented at 90 degrees contribute to enhanced thermal comfort. This observation is mainly attributed to the mean radiation temperature, as streets with North–South orientation mitigate harsh radiation, resulting in improved thermal conditions. For the"Strong heat stress"class, characterized by the numerical range of + 32 to + 38, the width of streets emerges as a significant and positively impactful feature. The primary reason remains similar to the previous class, as wider streets tend to elevate radiation levels, thereby creating conditions associated with higher UTCI index and lower thermal comfort. Figure 4 also depicts the"very Strong heat stress"class, encompassing the range of + 38 to + 46. In this class, the findings indicate that the 180-degree orientation emerges as the most significant positive factor for higher UTCI values. This orientation can block or significantly reduce airflow in an urban canyon. Reduced airflow leads to a lower rate of heat and pollutant dispersion, increasing the retention of heat in the streets and raising the UTCI values, which contributes to higher thermal discomfort. Conversely, the 90-degree orientation is identified as the next important feature, exhibiting a notable negative impact. This implies that while 180-degree orientations can lead to higher UTCI values and worse thermal comfort, 90-degree orientations tend to decrease UTCI, thus contributing positively to thermal comfort in the"Moderate heat stress"class.

Focusing on the PET index, Fig. 4 demonstrates that similar to the UTCI index, for the"Moderate heat stress"classification with a numerical range of + 29 to + 35, and the"extreme heat stress"class encompassing a range of > 40, 90-degree, and 180- degree orientations play a notably positive role as the most important feature, respectively. This suggests that while the 90-degree orientation enhances airflow and reduces solar exposure, leads to improving thermal conditions, the 180-degree orientation, on the other hand, traps heat and obstructs airflow, leading to adverse thermal conditions and a potential buildup of pollutants. It is possible that the “strong heat stress” classification represents a middle ground with less definitive results in terms of absolute positive and negative impacts. To facilitate a more comprehensive interpretation of the results within this class, it may be beneficial to explain these features in relation to previous and subsequent classes, where they are associated with clearly defined positive and negative thermal conditions, respectively.

Considering the overall results of feature rankings, the PMV index appears slightly different from the UTCI and PET indices. However, upon closer examination of its classes, it becomes apparent that the feature priorities and their respective positive and negative impacts on thermal comfort within each class closely resemble those observed in the UTCI index. For instance, the results of the"warm"classification, with the numerical range of + 2 to + 3 align closely with the"Moderate heat stress"class of the UTCI index. Accordingly, 90-degree orientations have a notably positive effect as the most influential feature in this class. Similarly, the “hot” class with a numerical range of + 3 to + 4 aligns closely with the"Strong heat stress"class of UTCI, in which the width of streets emerges as a significant and positively impactful feature. Finally, the “very hot” classification, as the last class of PMV with a numerical range of > + 4 shows similarity with the"very Strong heat stress"class of UTCI. Based on the findings of this class, the 180-degree orientation emerges as the most significant positive factor, which contributes to higher PMV and lower thermal comfort.

## Discussion

The quality of the input dataset plays a critical role in determining the performance and reliability of machine learning models, particularly in the classification of OTC levels. The dataset used in this study has been carefully curated to ensure both representativeness and robustness in urban climate analysis. Previous research has predominantly concentrated on examining the influence of various morphological factors on predicting and enhancing OTC conditions (Tehrani et al. 2024; Wu et al. 2023).However, there is a insufficiency of studies that simultaneously consider morphological factors on a large scale. This study intended to address this gap by comprehensively examining a wide range of morphological factors through extensive field measurements, focusing on 173 streets and 1168 study points. The data collection process was lengthy and challenging, but resulted in a meticulous dataset encompassing diverse orientations, H/W ratios, street widths, building heights, and the number of trees for each study point. Furthermore, while some previous studies have focused on specific OTC metrics (Narimani et al. 2022; Zadeh et al. 2022) this study adopted a concurrent approach by selecting three different OTC indices based on measured data. This thorough consideration facilitated the creation of a comprehensive dataset as an input file for machine learning models.

To further enhance the reliability of the dataset, extensive data validation and optimization techniques have been employed. The collected variables were assessed for completeness and consistency, ensuring that missing values and measurement errors were minimized. By integrating these considerations, the dataset not only improves ML classification performance but also supports data-driven urban design decisions aimed at enhancing OTC.

### ML Optimization and best model selection

In this study, employing BO techniques to optimize machine learning models for predicting OTC across various scenarios, provides a different perspective. Through meticulous exploratory analyses and hyperparameter tuning, the performance metrics including weighted accuracy, recall, precision, and F1-Score were evaluated for each algorithm across the three scenarios. In line with previous research (Jeong et al. 2022; Wang et al. 2023) current findings highlight the efficiency of ensemble methods such as XGBoost in accurately forecasting PMV. This is consistent with established literature that emphasizes the strength of ensemble methods in capturing non-linear relationships and intricate patterns within datasets (Karimi et al. 2024; Zhou et al. 2024).Similarly, the success of the CatBoost in forecasting the UTCI underscores the significance of gradient boosting techniques, particularly in handling categorical variables effectively. This resonates with recent advancements in gradient boosting algorithms, as exemplified by the work of (Guo et al. 2024a, b) and (Yang et al. 2023), who demonstrated the superior performance of CatBoost in various classification tasks. Moreover, In line with previous research (Bhattacharya and Mishra 2018; Davies et al. 2018) our findings underscore the growing significance of RF models in OTC research. In the context of OTC, RF models offer several advantages, including their ability to handle non-linear relationships and interactions among various environmental variables, such as street orientation, vegetation cover, and surface materials. Additionally, the ensemble nature of RF models, which aggregates predictions from multiple decision trees, enhances their robustness and generalization to unseen data, thereby improving their efficacy in accurately predicting PET values. This aligns with prior research (Qian et al. 2024) indicating the suitability of RF models for environmental modelling tasks, such as land use classification (Zhang et al. 2019), air quality prediction(López et al. 2023), and now, OTC assessment.

However, the rather underperformance of the KNN algorithm in comparison with other algorithms prompts a critical re-evaluation of its suitability in the context of PET and PMV prediction. Despite its intuitive appeal and simplicity, KNN's inability to capture complex relationships inherent in PET and PMV index raises questions regarding its applicability, highlighting the need for more sophisticated methods capable of discerning intricate patterns within multi-dimensional urban microclimate. This deviation from prior studies, such as those by Pantavou et al. (2022) (Pantavou et al. 2022) and Fard et al. (2022) (Fard et al. 2022), underscores the importance of considering algorithmic nuances and dataset-specific characteristics in model selection. Likewise, the suboptimal performance of the SVM model in UTCI prediction challenges the prevailing perception of SVM's efficacy in classification tasks. This discrepancy may be attributed to suboptimal parameter tuning or the complex interplay between kernel functions and regularization parameters, as discussed by various researchers in the field of ML optimization (Hamed et al. 2024; Liu et al. 2022a, b).

### Interpretability of the model

The integration of SHAP values for feature importance ranking in the current research provides a thorough comprehension of the morphological factors shaping OTC across diverse scenarios. By juxtaposing these findings with existing research, we can discern both confirmatory trends and novel insights, thereby enhancing our grasp of OTC dynamics within urban contexts.

Within the UTCI and PET metrics, the pronounced influence of the NS (90-degree) orientation underscores its pivotal role in regulating outdoor thermal environments. This observation resonates with previous studies(Park et al. 2014)emphasizing the importance of street orientation in mitigating heat stress and optimizing thermal comfort. Street orientations perpendicular to prevailing solar angles, such as the 90-degree orientation, can significantly modulate solar exposure and shading patterns, thus affecting surface temperatures and overall OTC circumstances (Ali-Toudert and Mayer 2007). Conversely, prolonged exposure to direct solar radiation, characteristic of streets oriented EW (180 degrees), can lead to higher surface temperatures and increased heat absorption, contributing to elevated ambient air temperatures within the street canyon. Moreover, the limited natural shading experienced by streets oriented at 180 degrees exacerbates heat build-up and thermal discomfort for pedestrians and residents, as surfaces like roadways and buildings absorb and hold heat without adequate shade. Additionally, the orientation of streets at 180- degrees can disrupt airflow patterns within the urban environment, potentially hindering the dispersion of heat and pollutants. Consequently, streets oriented EW may experience reduced ventilation and airflow compared to NS oriented streets, further exacerbating thermal discomfort and air quality issues. These factors collectively contribute to poorer outdoor thermal comfort conditions associated with streets oriented at 180- degrees, aligning with previous research (Ali-Toudert and Mayer 2006) highlighting the detrimental effects of solar exposure and airflow dynamics on OTC.

The feature importance rankings within the PMV index offer both novel insights and potential challenges when compared to previous research. The unexpected prominence of street width as the most influential factor diverges from anticipated trends, highlighting the nuanced nature of PMV modelling and the importance of contextual specificity. While previous studies have predominantly focused on factors like street orientation (Delpak et al. 2021; Guo et al. 2019; Narimani et al. 2022), the recognition of street width's significance enriches our understanding of urban thermal comfort dynamics. However, this deviation raises questions about the consistency and generalizability of existing thermal comfort models, challenging established norms in the field. The prominence of street width can be attributed to its influence on urban microclimate dynamics, including airflow patterns and solar exposure, which contribute to improved wind corridors and mitigated solar radiation in narrow streets. Additionally, it is necessary to mention that, upon closer examination of the categorized sub-classes for the PMV index, it becomes evident that orientation significantly influences thermal comfort levels. Specifically, the sub-class characterized by a 90-degree orientation exhibited the most favourable thermal comfort conditions; while, the dominance of a 180-degree orientation correlated with less favourable thermal comfort conditions. This observation aligns with findings from other indices within the study, indicating the pivotal role of orientation in shaping thermal comfort outcomes.

The findings underscore the importance of orientation in determining microclimatic conditions, with a 90-degree orientation contributing to enhanced thermal comfort, whereas a 180-degree orientation tends to exacerbate thermal circumstances. These findings underscore the necessity for nuanced modelling approaches that account for the diverse array of factors shaping outdoor thermal environments. Overall, while the emergence of street orientation and width as significant factors present valuable insights, additional investigation is needed to validate and refine existing modelling frameworks, advancing our understanding of OTC dynamics and informing effective strategies for heat stress mitigation in urban backgrounds.

### Varied response of OTC indices to urban morphology

The observed variations in how UTCI, PET, and PMV respond to urban morphological conditions can be attributed to the distinct physiological and environmental parameters prioritized by each index, as well as their computational frameworks. These differences are further amplified by the machine learning models’ ability to capture non-linear relationships between features and outcomes.

In this regard, UTCI’s holistic integration of radiant heat, wind speed, humidity, and air temperature makes it particularly sensitive to street orientation and H/W ratio. For instance, 90° (N-S) orientations reduce direct solar exposure by casting longer shadows, thereby lowering mean radiant temperature (Tmrt), a dominant driver of UTCI. Conversely, 180° (E-W) orientations maximize solar gain during peak hours, elevating Tmrt and UTCI values. This aligns with studies demonstrating that orientation-driven solar exposure significantly impacts radiant heat in urban canyons (Ali-Toudert and Mayer 2006; Johansson et al. 2014). The CatBoost model’s superior performance in predicting UTCI further underscores its ability to handle categorical variables like orientation, which are critical for capturing radiant heat dynamics. On the other hand, PET incorporates human thermoregulation parameters (e.g., metabolic rate, clothing insulation), making it highly responsive to shading and solar radiation. Wider streets reduce shading, increasing solar exposure and PET values, while narrower streets with higher H/W ratios enhance shading and lower PET. This explains why H/W ratio ranks higher in PET’s feature importance compared to UTCI. The RF model’s strength in capturing hierarchical interactions between variables likely contributed to its superior PET prediction accuracy. These findings resonate with prior work emphasizing the role of urban geometry in modulating physiological heat stress (Fang et al. 2018; Fröhlich et al. 2019). Turning to PMV, this index adapted for outdoor use via the Klima-Michel-Modell, places greater emphasis on wind speed and airflow patterns. Wider streets disrupt wind corridors, reducing ventilation and increasing heat stagnation, which elevates PMV values. Conversely, narrower streets enhance wind acceleration, improving thermal comfort. The prominence of street width in PMV’s SHAP rankings aligns with microclimate studies linking urban geometry to airflow dynamics (Delpak et al. 2021; Guo et al. 2019). The XGBoost model’s ability to model non-linear relationships likely amplified its sensitivity to street width’s role in modulating wind speed, contributing to its superior PMV prediction performance. By clarifying these index-specific mechanisms, urban planners can better prioritize design strategies for their specific aims and designs.

### Limitations and future studies

The current study has potential limitations that could affect the breadth and applicability of its findings. Firstly, While the current study is conducted in a cold semi-arid climate, the methodological framework is designed to be adaptable to other climatic and geographical contexts. Future research should validate the framework in cities with diverse climates (e.g., tropical, temperate, and arid) to assess its transferability and identify region-specific trends. Additionally, incorporating climatic and geographical variables as input features could enhance the framework's general applicability, providing urban planners with a versatile tool for optimizing outdoor thermal comfort across different regions. On the other hand, to address the seasonal limitation of current study, future research will focus on expanding data collection to encompass multiple seasons. This will enable a comprehensive evaluation of seasonal variations in OTC, particularly in colder months when cold stress may influence pedestrian comfort and urban design strategies. It is worth noting that due to vast aspects of vegetation studies, current research focused merely on number of trees to consider the effects on climatic factors. Obviously different characteristics would provide more precise results (such as tree type, crown size, etc.).

In addition, the reliability of the results may be influenced by the quality and accessibility of the data utilized, as well as potential limitations in the comprehensiveness of the datasets on outdoor thermal comfort. Moreover, while the study evaluated various performance metrics for assessment, there could be additional metrics that might offer a more inclusive understanding of model performance. For example, incorporating sophisticated data preprocessing methods such as Principal Component Analysis (PCA) and Minimum Noise Fraction (MNF) can improve feature selection and reduce dimensionality, ultimately leading to more reliable OTC classification. These approaches can refine predictive performance and contribute to the development of more accurate and efficient thermal comfort assessment models. In adition, exploring alternative machine learning models and optimization techniques could provide insights into their comparative performance and robustness. Lastly, considering social characteristics like population density and socio-economic status could enrich our comprehension of OTC and inform equitable urban planning strategies. In this regard, validating the findings through interviews and other subjective assessments could strengthen the practical applicability of the research outcomes.

## Conclusion

The study conducted an extensive examination of six distinct machine learning models optimized through Bayesian optimization techniques across three key OTC indices: UTCI, PET, and PMV. Through meticulous exploratory analyses and hyperparameter tuning, the performance metrics including weighted accuracy, recall, precision, and F1-Score were evaluated for each model across the three scenarios. Despite observing marginal differences in model performance across the indices, notable trends emerged.

1. CatBoost Classifier with BO exhibited superior performance for UTCI prediction, while the RF classifier integrated with BO excelled in PET estimation, and the XGBoost Classifier combined with BO demonstrated optimal performance for PMV prediction. This knowledge could help researchers to focus on the most proficient models for each OTC metrics and consider optimization process as an essential part of ML predictions.
2. Beyond model performance, the study investigates the influence of morphological features on OTC across three thermal indices: UTCI, PET, and PMV, utilizing SHAP values to prioritize the importance of different features. Consistently, the 90-degree orientation, width of streets, and 180-degree orientation emerged as the most influential factors influencing OTC. These outcomes provide urban planners and policymakers with actionable insights to optimize outdoor environments for enhanced thermal comfort.
3. Through the meticulous analysis of binary SHAP values, this study also unveils intricate relationships between various urban features and thermal comfort indices. In the UTCI index analysis, orientations of streets stand out as pivotal factors shaping thermal comfort across different heat stress classifications. Notably, 90-degree orientations consistently contribute positively to moderate heat stress conditions by mitigating harsh radiations, while 180-degree orientations exacerbate thermal discomfort in very strong heat stress scenarios. Similarly, in PET index assessments, 90-degree orientations demonstrate a positive role in moderating thermal stress, contrasting with the adverse effects of 180-degree orientations. The PMV index, although slightly different in feature prioritization, mirrors the trends observed in UTCI and PET indices across its classifications.

These findings underscore the critical effect of urban morphology on OTC, emphasizing the importance of urban planning strategies geared towards optimizing environmental conditions for inhabitants.

## Supplementary Information

Below is the link to the electronic supplementary material.

Supplementary file1 (DOCX 459 KB)

## Data Availability

The data presented in this study are available on request from the corresponding author.
