# Supplementary material for: A measurement-based framework integrating machine learning and morphological dynamics for outdoor thermal regulation
Source: Int J Biometeorol. 2025 Apr 21;69(7):1645–62. doi: 10.1007/s00484-025-02921-8 (PMC12179017; doi:10.1007/s00484-025-02921-8)
Supplement: Supplementary file 1 — Supplementary file1 (DOCX 459 KB) [file 484_2025_2921_MOESM1_ESM.docx]

# **Appendix**

**Appendix 1.** Monthly Air Temperature (Ta) Variation in Tabriz from 2012 to 2022


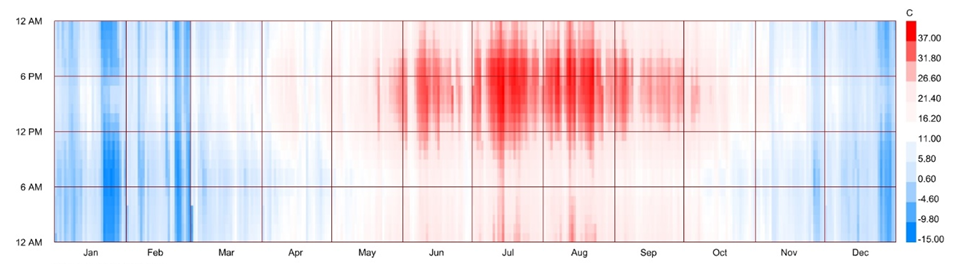


**Appendix 2**. Measured parameters and features of the instruments used in the research

| **Instrument** | **Measured parameter** | **Accuracy** | **Range** |
| --- | --- | --- | --- |
| Globe-thermometer | Tg, Ws | 0.1 °C ° C at 0 °C DIN 43760 1/3 | 0.1 |
| Weather data logger | Ta | ±0.9 °C from 40 °C to 60 °C | −40 °C–70 °C |
|  |  | ±0.5 °C from 5 °C to 40 °C |  |
|  |  | ±1.1 °C from −20 °C to 5 °C ± 3% |  |
|  | Rh | ±3% | 0 to 90℅ |

**Appendix 3.** Definition and Hyperparameters of Various ML Algorithms

| Algorithms | Definition | Hyperparameters |
| --- | --- | --- |
| KNN | K-Nearest Neighbors (KNN) model classifies data by assessing the distances between samples. it makes arrangement in results by matching the closest neighboring models. | ‘leaf_size’, ‘N_neighbors', ‘P' |
| DT | Decision Tree (DT) is a forecasting technique, which utilizes a tree-like organisation to decide according to characteristics. this enables using hierarchical options to represent possible results. | ‘Max_depth’, ‘min_samples_leaf','Min_samples_split' |
| SVM | Support Vector Machines (SVM) is a strong supervised algorithm that determines ideal decision borders through increasing the boundary between different classifications. It classifies datasets efficiently and handles non-linear relations using kernel functions. | ‘C','degree','kernel' |
| RF | Random Forest (RF) is a method in collaborative learning that combines various decision trees to improve the precision of forecast, mitigate over-fitting, and decrease intricate relations within datasets. | ‘min_samples_split','n_estimators' |
| XGBOOST | It is a gradient boosting method that chronologically merges feeble learners, like DT. It uses optimizing systems to obtain higher analytical precision and manage intricate affairs inside datasets. | ‘colsample_bytree','gamma','min_child_weight','learning_rate','max_depth','r |
| CATBOOST | CatBoost stands out as an advanced machine learning method designed to optimize boosting algorithms, specifically crafted to manage categorical attributes. It results in enhanced predictive precision, particularly in datasets comprising such variables. | ‘depth','iterations','l2_leaf_reg','learning_rate' |

**Appendix 4.** Various algorithms combined with (BO) and the resulting optimal hyperparameters for each OTC index

| Algorithm | OTC units | Best Hyperparameters |
| --- | --- | --- |
| KNN + BO | PMV | 'n_neighbors': 1.003 |
|  | PET | 'n_neighbors': 5.358 |
|  | UTCI | 'n_neighbors': 11.427 |
| DT+ BO | PMV | 'max_depth': 11.427, 'min_samples_leaf': 7.436, 'min_samples_split': 12.849 |
|  | PET | 'max_depth': 7.971, 'min_samples_leaf': 1.591, 'min_samples_split': 10.830 |
|  | UTCI | 'max_depth': 11.427, 'min_samples_leaf': 7.436, 'min_samples_split': 12.849 |
| SVM+ BO | PMV | 'C': 6.031, 'gamma': 0.545 |
|  | PET | 'C': 9.636, 'gamma': 0.384 |
|  | UTCI | 'C': 5.492, 'gamma': 0.715 |
| RF+ BO | PMV | 'max_depth': 20.0, 'min_samples_leaf': 1.0, 'min_samples_split': 4.698, 'n_estimators': 115.639 |
|  | PET | 'max_depth': 20.0, 'min_samples_leaf': 1.0, 'min_samples_split': 2.0, 'n_estimators': 85.257 |
|  | UTCI | 'max_depth': 11.884, 'min_samples_leaf': 9.335, 'min_samples_split': 8.027, 'n_estimators': 102.961 |
| XGBOOST+ BO | PMV | 'colsample_bytree': 0.898, 'learning_rate': 0.151, 'max_depth': 9.912, 'min_child_weight': 4.680, 'n_estimators': 120.129, 'subsample': 0.864 |
|  | PET | 'colsample_bytree': 0.889, 'learning_rate': 0.262, 'max_depth': 9.850, 'min_child_weight': 8.192, 'n_estimators': 515.331, 'subsample': 0.890 |
|  | UTCI | 'colsample_bytree': 0.559, 'learning_rate': 0.195,  'max_depth': 4.003, 'min_child_weight': 9.502, 'n_estimators': 569.663, 'subsample': 0.707 |
| CATBOOST+ BO | PMV | 'depth': 4.121, 'iterations': 174.892, 'l2_leaf_reg': 8.00, 'learning_rate': 0.262 |
|  | PET | 'depth': 7.408, 'iterations': 188.839, 'l2_leaf_reg': 1.639, 'learning_rate': 0.035 |
|  | UTCI | 'depth': 6.957, 'iterations': 71.210, 'l2_leaf_reg': 9.556, 'learning_rate': 0.112 |

**Appendix 5.** Definitions and equations used to determine performance metrics within each category

| Performance Metrics | Definitions | Equation Number | Equations |
| --- | --- | --- | --- |
| Accuracy | The percentage of accurately predicted samples inside the specific category is known as the "accuracy" of that category and can be determined by Equation 3. Which 𝑇𝑃 represents true positive; 𝑇𝑁 is true negative, 𝐹𝑃 is false positive; and 𝐹𝑁 is false negative | Eq. 3 | $Accuracy=\frac{TP + TN}{TP + FP + TN + FN}$ |
| Precision | The "Precision" of each category is referred to the F of accurately predicted samples inside the subset of samples that obtain a positive result for that particular category. | Eq. 4 | $Precision=\frac{TP}{TP + FP}$ |
| Recall | Recall for each category is the fraction of accurately anticipated samples inside the subset of samples which are genuinely positive for that category | Eq. 5 | $Recall=\frac{TP}{TP + FN}$ |
| F1 Score | The "F1 score" for each category offers a thorough evaluation of the model's recall and accuracy. A larger F1 score indicates higher adaptation capacity of the model, which is closer to 1. | Eq. 6 | $F1 score=2 \times\frac{Precision \times Recall}{Precision + Recall}$ |

**Abbreviation**

| BO | Bayesian Optimization |
| --- | --- |
| CatBoost | Categorical Boosting |
| Clo | Clothing Thermal Resistance |
| DT | Decision Tree |
| H | Height |
| H/W | Height to Width ratio |
| ISO | International Organization for Standardization |
| KNN | K-Nearest Neighbor |
| NT | Number of Trees |
| OR | Orientation |
| PET | Physiologically Equivalent Temperature (°C) |
| PMV | Predicted Mean Vote |
| RF | Random Forest |
| RH | Relative Humidity (%) |
| SHAP | SHapley Additive xPlanations |
| SVM | Support Vector Machine |
| Ta | Air Temperature (°C) |
| Tg | Globe Temperature (°C) |
| Tmrt | Mean Radiant Temperature (°C) |
| UTCI | Universal Thermal Climate Index (°C) |
| V | Wind Speed (m/s) |
| W | Width |
| XGBoost | Extreme Gradient Boosting |
